# Characterization of paucibacillary ileal lesions in sheep with subclinical active infection by *Mycobacterium avium* subsp. *paratuberculosis*

**DOI:** 10.1186/s13567-018-0612-0

**Published:** 2018-12-04

**Authors:** Salvatore Pisanu, Tiziana Cubeddu, Carla Cacciotto, Ylenia Pilicchi, Daniela Pagnozzi, Sergio Uzzau, Stefano Rocca, Maria Filippa Addis

**Affiliations:** 1 Porto Conte Ricerche, SP 55 Porto Conte/Capo Caccia, Km 8.400, Loc. Tramariglio, 07041 Alghero, Italy; 2 Dipartimento di Medicina Veterinaria, Università degli Studi di Sassari, Via Vienna 2, 07100 Sassari, Italy; 3 Dipartimento di Scienze Biomediche, Università degli Studi di Sassari, Viale S. Pietro 43/B, 07100 Sassari, Italy; 4 Dipartimento di Medicina Veterinaria, Università degli Studi di Milano, Via Celoria 10, 20133 Milan, Italy

## Abstract

Paratuberculosis (PTB) or Johne’s disease is a contagious enteritis of ruminants caused by *Mycobacterium avium* subsp. *paratuberculosis* (MAP). Ovine PTB is less understood than bovine PTB, especially concerning paucibacillary infection and its evolution into clinical disease. We combined shotgun proteomics, histopathology and immunohistochemistry for the characterization of ileal tissues collected from seven asymptomatic sheep negative to serum ELISA, positive to feces and tissue MAP IS900 and F57 PCR, histologically classified as paucibacillary, actively infected, together with 3 MAP-free controls (K). Following shotgun proteomics with label-free quantitation and differential analysis, 96 proteins were significantly changed in PTB vs K, and were mostly involved in immune defense processes and in the macrophage-MAP interaction. Principal component analysis (PCA) of protein abundances highlighted two PTB sample clusters, PTB1 and PTB2, indicating a dichotomy in their proteomic profiles. This was in line with the PCA of histopathology data and was related to features of type 2 (PTB1) and type 3a (PTB2) lesions, respectively. PTB2 proteomes differed more than PTB1 proteomes from K: 43 proteins changed significantly only in PTB2 and 11 only in PTB1. The differential proteins cathelicidin, haptoglobin, S100A8 and S100A9 were evaluated by immunohistochemistry. K tissues were negative to cathelicidin and haptoglobin and sparsely positive to S100A8 and S100A9. PTB tissues were positive to all four proteins, with significantly more cells in PTB2 than in PTB1. In conclusion, we described several pathways altered in paucibacillary PTB, highlighted some proteomic differences among paucibacillary PTB cases, and identified potential markers for disease understanding, staging, and detection.

**Electronic supplementary material:**

The online version of this article (10.1186/s13567-018-0612-0) contains supplementary material, which is available to authorized users.

## Introduction

Paratuberculosis (PTB) or Johne’s disease is a chronic, contagious enteritis of ruminants caused by *Mycobacterium avium* subspecies *paratuberculosis* (MAP). PTB is especially relevant in farmed ruminants [1–4] for the economic consequences caused by increase in mortality, decrease in milk production, and weight loss [5, 6]. In addition, viable MAP can be found in pasteurized milk and milk products with a potential risk of zoonotic transmission [7–9].

MAP and its role in PTB have been the subject of numerous studies on disease progression and evolution in cattle. Our knowledge in this respect has increased considerably, but some aspects remain unclear also in this species, and more effective tools for disease diagnosis and control are still needed [10]. MAP transmission can occur by the fecal–oral route, in utero, and by ingestion of contaminated colostrum or milk [10–15]. Once the bacterium reaches the intestine, it is taken up by M cells and translocated across the intestinal mucosa, where it is internalized by naive macrophages and can lead to persistent infection [16]. In cattle, the disease typically goes from an early subclinical, paucibacillary phase to a later multibacillary phase with severe clinical manifestations of the disease that include wasting and profuse, watery diarrhea [10]. In sheep, PTB is more insidious and classification is less clearly defined [2, 17], leading to a great underestimation of its worldwide diffusion. According to several authors, the largest number of sheep in an affected flock are infected but asymptomatic [18, 19]. These can either have a latent infection, encompassing the presence of MAP in tissues without signs of disease (subclinical infection), or an active infection, in which histological signs of the disease can be observed (subclinical disease) [20, 21]. A histological scoring system [22] classifies these signs into mild lesions represented by small focal granulomata of epithelioid cells limited to the Peyer patches (type 1) or extending to the adjacent mucosa (type 2), and more severe lesions with a multifocal cellular infiltration in mucosal areas not associated with lymphoid tissues and extending into the submucosa, with thickening of the mucosa and atrophy of villi (type 3a). According to Pérez and coworkers [22], symptomatic animals with the end-point disease (clinical disease) present two different types of lesion, lepromatous (type 3b) or tuberculoid (type 3c). Based on the degree of colonization, lesions can also be classified into paucibacillary, with few or no acid-fast bacilli (AFB), and multibacillary, with abundant AFB. Type 1, 2 and 3c lesions are paucibacillary, while type 3b are multibacillary. Type 3a lesions are mainly paucibacillary, but multibacillary patterns can also be present, indicating that a crucial “transition stage” may occur in animals with this type of lesions [22]. Actively infected sheep with the subclinical disease can either remain such for their whole life, acting as MAP reservoirs and shedders, or develop clinical disease by showing either paucibacillary (3c) or multibacillary (3b) lesions. At present, factors, pathways, stages and dynamics of disease progression are not completely understood [20], and the best diagnostic approach remains post-mortem evaluation with histopathology image analysis, which represents the best indicator to confirm PTB and define its stage [1, 2, 23, 24].

The availability of detailed information on the proteomic alterations introduced in ovine ileal tissues by the subclinical, paucibacillary, active MAP infection would provide novel information for understanding disease mechanisms, discover protein markers with potential for disease staging and, possibly, indicate host proteins that may be shed and detected in feces in vivo. This would also help experimental infection studies aimed at understanding PTB progression to clinical disease. Currently, however, proteomic information on MAP-infected tissues is limited to a preliminary study recently published by our research group and describing the changes occurring in the ileum of sheep with multibacillary, clinical PTB [25].

In this study, we combined histopathology, proteomics, and immunohistochemistry for the characterization of paucibacillary MAP lesions found in actively infected, subclinical sheep. Ileal tissues of asymptomatic, serum ELISA-negative, feces and tissue PCR-positive sheep were subjected to histopathological analysis and those classified as paucibacillary PTB were analyzed by shotgun proteomics in comparison to MAP-free controls. Differential proteins were investigated for host pathways activated during infection. Finally, proteins of interest were evaluated by immunohistochemistry in MAP-infected tissues and in MAP-free controls.

## Materials and methods

### Animals and tissues

Sheep ileal tissues used for the study belonged to a MAP-positive flock of Sarda sheep (*N* = 174). The flock was monitored by the farm veterinarian under an owner voluntary basis by clinical examination, evaluation of PTB symptoms, serological screening by ELISA for presence of anti-MAP antibodies in serum (IDEXX Laboratories, Inc., Westbrook, MA, USA), and IS900 and F57 PCR for presence of MAP in feces, as described previously [25]. Out of 174 sheep, 21 were both ELISA and PCR-positive, 20 were ELISA-positive and PCR-negative, and 15 were ELISA-negative and PCR-positive. The latter 15 were identified as those most probably affected by paucibacillary paraTBC. At routine slaughtering, the intestinal packages belonging to these 15 sheep (all females between 3 and 4 years of age) were retrieved at the slaughterhouse and brought to the Department of Veterinary Medicine at the University of Sassari for gross pathological anatomy examination. Separate aliquots of tissue from the proximal, intermediate, and distal ileum (*N* = 45 tissue samples) were collected from each sheep. Matched tissue aliquots were frozen at −80 °C or formalin-fixed and paraffin embedded for downstream molecular and histopathological characterization.

### Histopathological analysis and molecular characterization of ileal lesions

Three micrometre sections from paraffin-embedded tissue blocks from each ileal tract were subjected to haematoxylin–eosin and Ziehl–Neelsen (ZN) staining and examined to confirm presence of MAP and associated lesions. PCR was carried out on all 45 frozen tissues samples for confirmation of MAP infection, as described previously [25]. Briefly, DNA was isolated with the DNeasy^®^ Blood and Tissue kit (Qiagen, Hilden, Germany), and tested for presence of the MAP specific sequences IS900 and F57 by qualitative PCR. The MAP type was also characterized by IS1311, RFLP, and sequencing, as described previously [25, 26]. All 15 tested animals carried the sheep MAP strain (S). FFPE tissue sections obtained from the proximal, intermediate, and distal ileum of the 15 ELISA-negative, PCR-positive sheep (*N* = 45 tissue samples) were evaluated by histopathological analysis. Out of these 15 sheep, 7 showed histopathological lesions compatible with paucibacillary paraTBC and were selected for proteomic analysis (Additional file 1). Negative control tissues (K) were obtained from three sheep of a certified MAP-free flock. K sheep were negative to serum ELISA and feces PCR. Their intestinal tissues were negative to PCR, microscopical evaluation and Ziehl–Neelsen staining, and showed normal anatomy and histology.

### Tissue processing for protein extraction and shotgun proteomic analysis

Tissue sample preparation was carried out as previously described [25]. Briefly, 100 mg of tissue from the distal ileum of the 7 PTB and 3 K sheep (*N* = 10 tissue samples) was minced with a sterile knife, placed in Eppendorf safe-lock tubes (Eppendorf, Hamburg, Germany), immersed in extraction buffer and subjected to homogenization in a TissueLyser mechanical homogenizer (Qiagen, Hilden, Germany) followed by three cycles of sonication and freeze-thawing. Then, the extract was clarified by centrifugation, subjected to nucleic acid digestion, and protein concentration was determined. Extracts and residual pellets (*N* = 20) were processed by filter-aided sample preparation (FASP). Briefly, protein samples were subjected to reduction, alkylation, and trypsin digestion on Amicon Ultra-0.5 centrifugal filter units with Ultracel-10 membrane (Millipore, Billerica, MA, USA) to obtain sample digests for shotgun proteomic analysis. Peptide concentration of digests was determined by measuring absorbance at 280 nm with a NanoDrop 2000 spectrophotometer (Thermo Scientific, San Jose, CA, USA) using MassPREP *E. coli* Digest Standard (Waters, Milford, MA, USA) to create a calibration curve.

### Shotgun proteomic analysis of peptides

All peptide mixtures were analyzed by liquid chromatography-tandem mass spectrometry (LC–MS/MS) in duplicate runs (two technical replicates for each peptide mixture) on a Q-Exactive interfaced with an UltiMate 3000 RSLCnanoLC system (Thermo Scientific, San Jose, CA, USA), as described previously [27]. Four microgram of each peptide mixture were concentrated and washed onto a trapping precolumn (Acclaim PepMap C18, 75 µm × 2 cm nanoViper, 3 µm, 100 Å, Thermo Scientific) and fractionated on a C18 RP column (Acclaim PepMap RSLC C18, 75 µm × 50 cm nanoViper, 2 µm, 100 Å, Thermo Scientific) at a flow rate of 250 nL/min using a linear gradient of 245 min from 5 to 37.5% eluent B (0.1% formic acid in 80% acetonitrile) in eluent A (0.1% formic acid). Fragmentation occurred by higher energy collisional dissociation (HCD) and nitrogen as the collision gas. Proteome Discoverer (version 1.4; Thermo Scientific, Bremen, Germany) was used for protein identification using Sequest-HT as search engine. Each MS/MS spectrum was analyzed as follows. Database: *Bos taurus*, *Ovis aries*, and *Mycobacterium avium* downloaded from UniProtKB/Swiss-Prot (release 2017 06); enzyme: trypsin, with two missed cleavages allowed; precursor mass tolerance: 10 ppm; MS/MS tolerance: 0.02 Da; charge states: + 2, + 3, and + 4; cysteine carbamidomethylation as static modification and methionine oxidation as dynamic modifications. The percolator algorithm [28, 29] was used for assessing the significance of protein identification (*P* < 0.01) and for peptide validation (false discovery rate, FDR, < 0.01%) [28, 30]. Only rank 1 peptides and only proteins identified with at least two peptides and two spectral counts were considered. Peptide and protein grouping according to the Proteome Discoverer’s algorithm were allowed, applying the strict maximum parsimony principle. The protein list for each intestinal sample was built up by merging the data from the LC–MS/MS runs of the protein extract and of the residual pellet, generating 10 protein identification lists (1 for each sheep sample). For proteins having more than one entry, only those with the highest number of unique peptides and spectral counts (SpC) were selected for downstream analyses.

### Principal component analysis and hierarchical clustering of samples

Principal component analysis (PCA) and hierarchical clustering of samples according to proteomic results were carried out based on the normalized spectral abundance factor (NSAF) values of all identified proteins, obtained according to Old et al. [31], as an indicator of protein abundance [32]. PCA and hierarchical clustering of samples according to histopathological results were carried out based on the scores generated upon detailed histological assessment by two observers grading from 0 (absence of feature) to 3 (maximum degree of feature) and reported in Additional file 1. Data analysis was carried out with Perseus (v.1.6.0.7).

### Differential proteomic analysis

To estimate protein abundance and to compare the levels of the same protein among sample groups, a spectral counting approach was applied as described by Old et al. [31] and Zybailov et al. [33]. The R_SC_, that is the log_2_ ratio of protein abundances between two experimental groups, was calculated as described previously [34] and expressed as fold change (FC) [25]. Statistical significance of differences in protein abundance was assessed by the beta-binomial test with FDR correction by Benjamini-Hochberg [35, 36]. Only proteins with FC ≥ 2 or ≤ −2, with a *p*-value ≤ 0.05 and present in at least two samples of each group being compared were considered significant.

### STRING analysis

To investigate the biological role of differential proteins, their functional association at a system level [37] was assessed by performing a knowledge-based protein–protein interaction network analysis by means of the biological interface and web resource STRING (version 10.5) after replacing all *Ovis aries* UniProt IDs with the corresponding *Bos taurus* UniProt IDs. Specifically, KEGG enrichment analysis was aimed at mapping the differential proteins in biological pathways, while gene ontology (GO) analysis was aimed at mapping them into biological processes (BP), molecular functions (MF), and cellular components (CC).

### Immunohistochemistry

Immunohistochemistry (IHC) was carried out as described previously [38]. Antibodies and dilutions were as follows: rabbit anti-CAMP (Sigma-Aldrich), 1:1500; rabbit anti-S100A8 (Sigma-Aldrich), 1:1000; rabbit anti-haptoglobin (Thermo Fisher Scientific), 1:100; rabbit anti-S100A9 (Sigma-Aldrich), 1:1000. Signal evaluation was carried out by counting positive cells in 10 random fields at 200× magnification (PC). Statistical analysis of IHC results was carried out with GraphPad Prism version 5.03 for Windows (GraphPad Software, La Jolla, CA). According to the Shapiro–Wilk normality test the data followed a non-normal distribution, and a nonparametric Mann–Whitney U-test was therefore applied.

## Results

### Shotgun proteomic results, principal component analysis, and hierarchical clustering

**Figure 1:**
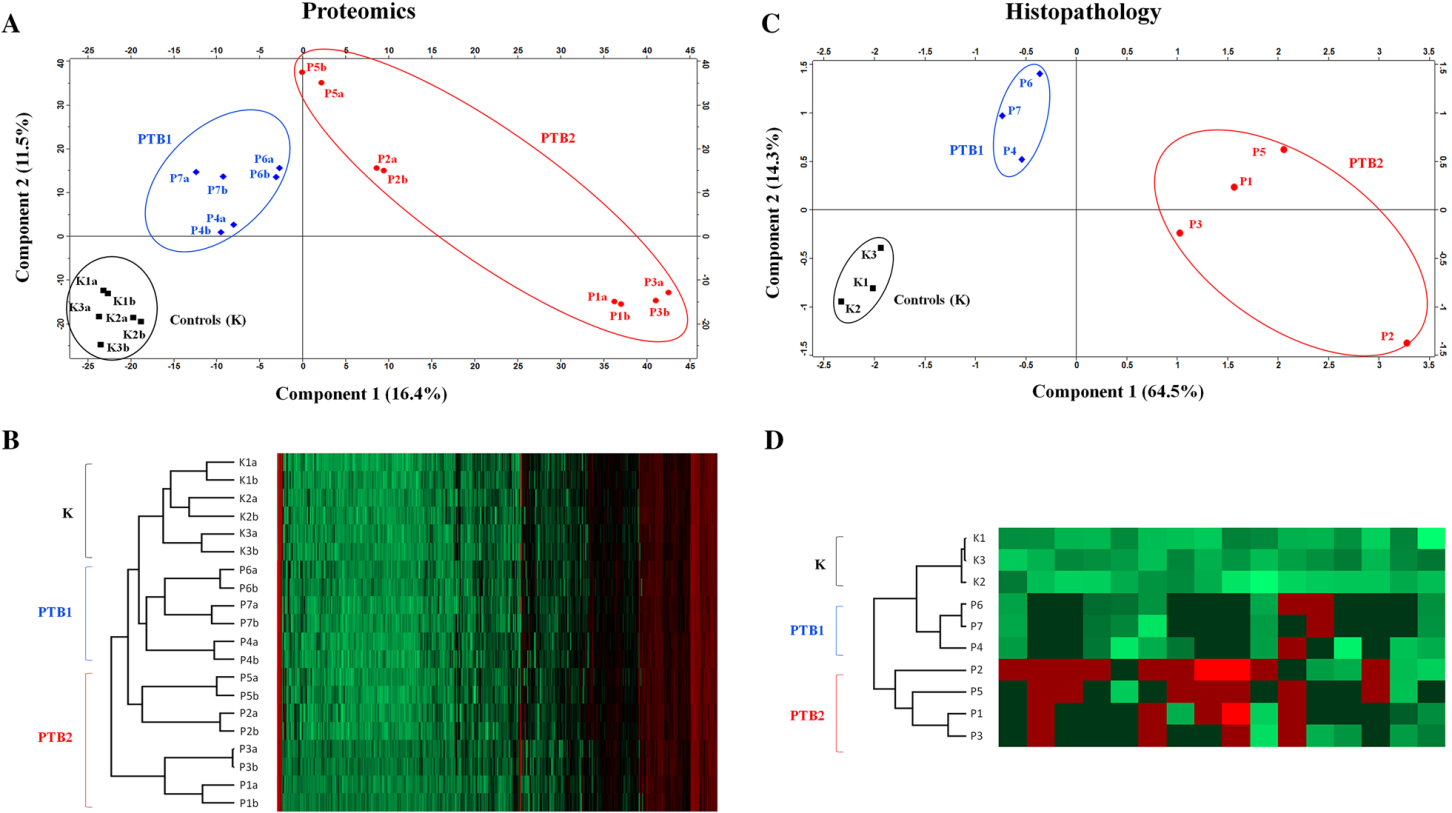
**Clustering of samples according to protein abundance values and histopathological features.** The two panels show the PCA clustering on top and the hierarchical clustering heatmap on the bottom. Left, clustering based on shotgun proteomics. Right; clustering based on histopathology. Two technical replicates were analyzed in shotgun proteomics experiments, and results are reported separately for the two replicates as a and b, respectively. P indicates paucibacillary samples; K indicates control samples. **A** Results of PCA based on protein abundance values in terms of normalized spectrum abundance factors (NSAFs). **B** Hierarchical clustering of samples based on NSAFs. Protein identities and abundances are reported in Additional file 3. **C** Results of PCA based on histopathological grading. **D** Hierarchical clustering of samples based on histopathological grading. Parameters and scores are detailed in Additional file 1.

A total of 2350 and 2292 proteins were identified in PTB and in K samples, respectively. Details on proteins, peptides, PSMs and search inputs are reported in Additional files 2 and 3. PCA and hierarchical clustering based on protein abundance values (NSAFs as reported in Additional file 3) generated three main clusters: one including all K samples, one including three PTB samples (P4, P6, and P7) and one including four PTB samples (P1, P2, P3, and P5) (Figures 1A and B). K and PTB1 were separated from PTB2 by the first component (16.4%), while K was separated from PTB1 by the second component (11.5%). PCA and hierarchical clustering based on a detailed histopathological analysis with grading scores (Additional file 1) produced similar sample clusters: one including K samples, one including P4, P7, and P6, and one including P1, P2, P3, and P5 (Figures 1C and D). K and PTB1 were separated from PTB2 by the first component (64.5%), while K was separated from PTB1 by the second component (14.3%). Therefore, PCA carried out on histopathological classification mirrored proteomic data and confirmed the PTB1/PTB2 clustering, characterized by a lower divergence of three samples (designated as PTB1 cluster) and a higher divergence of the other four (designated as PTB2 cluster) from controls (K). Based on histopathological observations, PTB1 samples presented features more compatible with the definition of type 2 lesions, while PTB2 samples were more compatible with the definition of type 3a lesions (Additional file 1).

### Differential proteomic analysis

In view of the results obtained by PCA and hierarchical clustering of both proteomics and histopathology results, the differential proteomic analysis was carried out by comparing: (i) all PTB samples vs K; (ii) only PTB1 samples vs K; and (iii) only PTB2 samples vs K. Results are reported in Table 1 and in Additional file 3.

**Table 1:** **Differential proteins in all PTB, PTB1 and PTB2 vs K samples, respectively**

Accession	Gene name	Description	All PTB vs K	PTB1 vs K	PTB2 vs K
P62808	H2B	Histone H2B type 1	***138.55***	***159.95***	***122.71***
P63048	UBA52	Ubiquitin-60S ribosomal protein L40	***70.26***	***67.05***	***72.67***
A0JNJ5	MYL1	Myosin light chain 1/3, skeletal muscle isoform	***16.55***	***23.39***	***11.42***
Q17QN3	SNRPN	Small nuclear ribonucleoprotein-associated protein N	***11.68***	***9.08***	***13.31***
A1L595	KRT17	Keratin, type I cytoskeletal 17	***11.29***	13.98^b^	***9.06***
*A0A0E2WA25*	*HtpG*	*Chaperone protein HtpG*	*8.67*	*5.32*	*11.19*
A2I7N0	SERPINA3-4	Serpin A3-4	***6.70***	***7.62***	***6.05***
Q9BE41	MYH2	Myosin-2	***5.76***	***5.37***	***5.78***
P22226	CATHL1A	Cathelicidin-1	***5.44***	***3.06***	***7.54***
Q29437	AOCX	Primary amine oxidase, liver isozyme	***5.29***	***5.69***	4.99^b^
Q56JX9	FABP2	Fatty acid-binding protein, intestinal	***5.07***	***9.92***	1.43^b^
Q3T104	SEC61G	Protein transport protein Sec61 subunit gamma	***4.69***	***4.21***	***5.28***
Q17QB3	ASAH1	Acid ceramidase	***3.75***	***3.11***	***4.17***
P19660	CATHL2	Cathelicidin-2	***3.67***	***2.14***	***5.10***
Q5E972	ORMDL2	ORM1-like protein 2	***3.28***	***2.69***	***3.73***
Q9N0K1	CD47	Leukocyte surface antigen CD47	***2.99***	1.98^b^	***3.40***
Q0IIG7	RAB5A	Ras-related protein Rab-5A	***2.89***	1.81^b^	***3.70***
P61625	ITGAL	Integrin alpha-L	***2.73***	−1.17^b^	***4.14***
Q1JPB0	SERPINB1	Leukocyte elastase inhibitor	***2.64***	1.28^b^	***3.72***
P85521	CD163	Scavenger receptor cysteine-rich type 1 protein M130	***2.56***	1.14^b^	***3.63***
Q58DS5	RAB13	Ras-related protein Rab-13	***2.50***	**2.33**	***2.40***
Q9BDK2	AIF1	Allograft inflammatory factor 1	***2.49***	1.13^b^	***3.51***
P79106	PAFAH2	Platelet-activating factor acetylhydrolase 2	***2.45***	2.05^b^	***2.76***
P09578	MT1C	Metallothionein-1C	***2.44***	**2.35**	2.43^b^
P83095	LACTB	Serine beta-lactamase-like protein LACTB	***2.44***	1.39^b^	***3.23***
Q95JC7	SLC1A5	Neutral amino acid transporter B (0)	***2.43***	2.05^b^	***2.72***
P55156	MTTP	Microsomal triglyceride transfer protein large subunit	***2.37***	***3.45***	1.56^a^
Q2YDN6	RPF2	Ribosome production factor 2 homolog	***2.37***	1.45^b^	***3.06***
P00766	CTRA	Chymotrypsinogen A	***2.21***	−***2.76***	***3.60***
Q28035	GSTA1	Glutathione S-transferase A1	***2.11***	***3.73***	−1.11^a^
Q599T9	MYD88	Myeloid differentiation primary response protein MyD88	***2.06***	***2.35***	***2.00***
P61955	SUMO2	Small ubiquitin-related modifier 2	***2.04***	1.94^b^	***2.12***
Q95115	STAT5A	Signal transducer and activator of transcription 5A	***2.04***	1.66^b^	***2.33***
Q3SWY9	RAB28	Ras-related protein Rab-28	1.73^b^	***2.42***	–
Q32L48	HIST1H2BN	Histone H2B type 1-N	1.55^b^	***2.25***	1.01^b^
A7YWK3	KRT73	Keratin, type II cytoskeletal 73	1.49^b^	***2.24***	−1.08^b^
A2VDL6	ATP1A2	Sodium/potassium-transporting ATPase subunit alpha-2	1.44^b^	***2.22***	−1.16^a^
Q2TBP0	PSMB7	Proteasome subunit beta type-7	1.64^a^	***2.10***	1.30^a^
P14568	ASS1	Argininosuccinate synthase	1.40^a^	***2.05***	−1.09^b^
Q2TBU0	HP	Haptoglobin	8.14^b^	–	***13.84***
P00639	DNASE1	Deoxyribonuclease-1	3.97^b^	−1.57^b^	***6.48***
P30922	CHI3L1	Chitinase-3-like protein 1	3.67^b^	1.16^b^	***5.80***
Q0P5C2	MTHFD2	Bifunctional methylenetetrahydrofolate dehydrogenase	3.04^b^	1.57^b^	***3.92***
Q1LZA3	ASNS	Asparagine synthetase	2.44^b^	1.25^b^	***3.53***
P00760	TRY1	Cationic trypsin	1.92^a^	2.33^b^	***3.03***
O77775	NCF2	Neutrophil cytosol factor 2	2.25^b^	1.20^b^	***3.03***
P08169	IGF2R	Cation-independent mannose-6-phosphate receptor	1.11^b^	−1.05^b^	***2.99***
P28782	S100A8	Protein S100-A8	1.94^b^	–	***2.89***
P32592	ITGB2	Integrin beta-2	1.86^b^	1.20^b^	***2.64***
Q2HJH7	MEMO1	Protein MEMO1	2.31^b^	1.85^b^	***2.62***
O75601	CASP13	Caspase-13	1.75^a^	1.01^a^	***2.60***
O77588	PLOD1	Procollagen-lysine,2-oxoglutarate 5-dioxygenase 1	1.91^b^	−1.12^b^	***2.57***
Q5EAD2	PHGDH	d-3-Phosphoglycerate dehydrogenase	1.98^a^	1.27^b^	***2.51***
A2VDZ9	VAPB	Vesicle-associated membrane protein-associated protein B	2.15^b^	1.72^b^	***2.46***
P31408	ATP6V1B2	V-type proton ATPase subunit B	1.12^b^	1.06^b^	***2.45***
P25326	CTSS	Cathepsin S	2.01^b^	1.15^b^	***2.38***
A5PJP1	BLOC1S3	Biogenesis of lysosome-related organelles complex 1 sub 3	1.76^b^	–	***2.38***
Q29RY9	NPL	*N*-acetylneuraminate lyase	1.74^a^	1.10^a^	***2.36***
A5PJN2	ERO1L	ERO1-like protein alpha	1.72^b^	1.06^b^	***2.30***
Q2TBK3	PLAC9	Placenta-specific protein 9	1.71^a^	1.02^b^	***2.23***
P31404	ATP6V1A	V-type proton ATPase catalytic subunit A	1.75^a^	1.11^b^	***2.22***
Q3ZBI6	FHL3	Four and a half LIM domains protein 3	1.70^a^	1.03^b^	***2.21***
Q3SZZ0	BRIX1	Ribosome biogenesis protein BRX1 homolog	1.88^b^	1.47^b^	***2.18***
O46563	ATP6V1H	V-type proton ATPase subunit H	1.62^b^	1.11^b^	***2.16***
P35541	SAA1	Serum amyloid A protein	1.41^b^	–	***2.15***
Q17QL5	TMEM30A	Cell cycle control protein 50A	1.82^b^	1.49^a^	***2.07***
Q865V6	CAPG	Macrophage-capping protein	1.56^b^	−1.10^b^	***2.05***
Q58D20	NLE1	Notchless protein homolog 1	1.90^b^	1.74^b^	***2.03***
P06868	PLG	Plasminogen	1.63^b^	1.17^b^	***2.00***
P80931	MCT1A	Mast cell protease 1A	−1.70^b^	1.19^b^	−***7.28***
Q6B410	LYSI	Lysozyme C	−1.92^b^	−1.07^b^	−***4.81***
Q3SZX4	CA3	Carbonic anhydrase 3	−1.74^b^	1.03^b^	−***4.29***
Q7YRZ7	GZMA	Granzyme A	−1.76^a^	−1.16^b^	−***2.91***
P19111	ALPI	Intestinal-type alkaline phosphatase	−1.28^b^	1.29^b^	−***2.62***
A8YXX7	TFF3	Trefoil factor 3	−1.43^b^	1.11^b^	−***2.54***
Q3T166	MPTX	Mucosal pentraxin	−1.01^a^	1.77^b^	−***2.52***
Q28106	CNTN1	Contactin-1	−1.40^b^	1.12^b^	−***2.43***
Q3MHR3	DYNLL2	Dynein light chain 2	−1.63^b^	−1.22^b^	−***2.16***
Q5EA62	FBLN5	Fibulin-5	−1.49^b^	−1.05^b^	−***2.16***
Q3MHN0	PSMB6	Proteasome subunit beta type-6	−1.63^b^	−1.30^b^	−***2.16***
Q3T0Z2	FABP6	Gastrotropin	−1.85^b^	−1.65^b^	−***2.05***
P31836	NCAM1	Neural cell adhesion molecule 1	−1.49^b^	−1.10^b^	−***2.02***
Q2M2T1	HIST1H2BK	Histone H2B type 1-K	−1.24^a^	−***3.55***	1.20^a^
Q3T052	ITIH4	Inter-alpha-trypsin inhibitor heavy chain H4	−0.83^a^	−***2.63***	1.82^b^
P61823	RNASE1	Ribonuclease, pancreatic	−1.11^a^	−***2.60***	1.66^a^
Q29RQ9	ORMDL1	ORM1-like protein 1	−1.59^b^	−***2.25***	−1.43^b^
A1A4Q4	TMA7	Translation machinery-associated protein 7	−1.00^a^	−***2.23***	1.41^b^
Q32PH8	EEF1A2	Elongation factor 1-alpha 2	−***2.01***	−1.56^a^	−**2.57**
Q0III9	ACTN3	Alpha-actinin-3	−***2.36***	−***2.54***	−***2.24***
P00978	IATR	Inter-alpha-trypsin inhibitor	−***4.09***	−***4.38***	−***3.37***
Q9BE39	MYH7	Myosin-7	−***6.04***	−***6.80***	−***5.23***
Q9BE40	MYH1	Myosin-1	−***6.28***	−***7.08***	−***5.44***
O62664	PTGS1	Prostaglandin G/H synthase 1	−***6.39***	−***7.20***	−***5.54***
Q58DW4	SNRPB	Small nuclear ribonucleoprotein-associated protein B’	−***6.81***	−***7.66***	−***5.90***
Q32KN8	TUBA3	Tubulin alpha-3 chain	−***52.20***	−***60.66***	−***46.68***
Q2HJ86	TUBA1D	Tubulin alpha-1D chain	−***97.29***	−***113.10***	−***87.02***

Differences in abundance are reported as fold changes in the respective sample comparisons. Statistically significant proteins are in bolditalics. The only statistically significant MAP protein is in italics.

In bolditalics: fold change ≤ −2 or ≥ 2, beta-binomial test *P* ≤ 0.05.

^a^Fold change ≥ −2 or ≤ 2, beta-binomial test *P* ≤ 0.05

^b^Any fold change with non-significant beta-binomial test.

A total of 96 proteins showed statistically significant differences (Table 1). Of these, 69 were higher and 27 were lower in PTB vs K, respectively. Of the 69 increased proteins, 33 were statistically significant in all PTB samples, 6 only in PTB1, and 30 only in PTB2. Of the 27 decreased proteins, 9 were statistically significant in all PTB samples, 5 only in PTB1, and 13 only in PTB2 (Table 1). The higher number of differential proteins detected in PTB2 samples indicates that these differ more than PTB1 samples from K, and suggests the presence of two different levels of tissue involvement in the PTB sample set. Eleven proteins were identified in the MAP database and included in the differential analysis (Additional file 3). Among them, only chaperone protein HtpG passed the thresholds for FC value and statistical significance (Table 1).

### KEGG enrichment and gene ontology analysis of differential proteins

Differential proteins were evaluated for protein networks by STRING. KEGG enrichment and GO results are summarized in Additional file 4.

**Figure 2:**
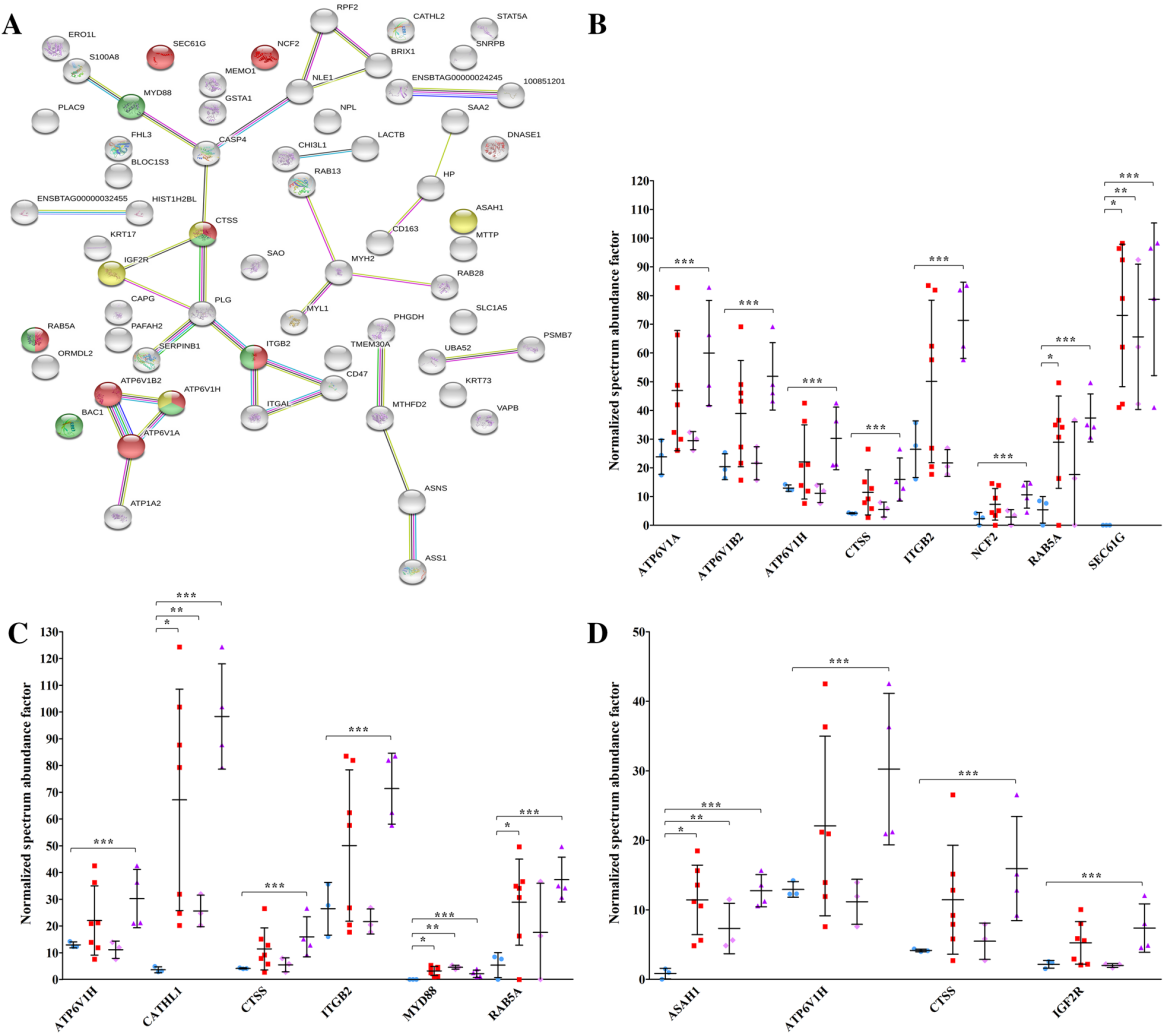
**KEGG enrichment analysis of proteins significantly increased in PTB vs K.** The figure reports the protein network and the proteins included in each category, with the respective abundance increases in all PTB samples, in PTB1, and in PTB2, respectively. **A** Protein network according to STRING. Proteins associated with phagosome, lysosome, and tuberculosis are indicated in red, green, and yellow, respectively. Seven different colored lines link nodes and represent seven types of evidence used in predicting associations. Green lines: neighborhood evidence; red lines: presence of fusion evidence; blue lines: co-occurrence evidence; black lines: co-expression evidence; purple lines: experimental evidence; light blue lines: database evidence; yellow lines: text-mining evidence. Plots show the relative abundances of proteins in the phagosome (**B**), lysosome (**C**), and tuberculosis (**D**) pathways. Plots report values obtained in K (sky blue), PTB (red), PTB1 (pink), and PTB2 (purple) samples. Asterisks indicate statistically significant differences between PTB vs K (*), PTB1 vs K (**), and PTB2 vs K (***) according to the t-test (*p* value ≤ 0.05). For protein identities corresponding to gene names, please see Table 1.

For the 69 proteins increased in all PTB samples, KEGG enrichment (Figure 2A) highlighted three main networks. The first network was phagosome (*P* = 5.97E−06), including V-type proton ATPase catalytic subunit A (ATP6V1A), V-type proton ATPase subunit B (ATP6V1B2), V-type proton ATPase subunit H (ATP6V1H), cathepsin S (CTSS), integrin beta-2 (ITGB2), neutrophil cytosol factor 2 (NCF2), ras-related protein Rab-5A (RAB5A), and protein transport protein Sec61 subunit gamma (SEC61G) (Figure 2B). The second network was tuberculosis (*P* = 1.03E−03), including ATP6V1H, cathelicidin 1 (CATHL1A), CTSS, ITGB2, myeloid differentiation primary response protein MyD88 (MYD88), and RAB5A (Figure 2C). And the third network was lysosome (*P* = 2.82E−02), including acid ceramidase (ASAH1), ATP6V1H, CTSS, and IGF2R (Figure 2D). Figures 2B, C and D report the relative abundance (NSAF) of the proteins that describe the pathways. Eight out of these 11 proteins (ATP6V1A, ATP6V1B2, ATP6V1H, CTSS, IGF2R, ITGB2, NCF2, and RAB5A) were significantly higher only in PTB2. Only 4 proteins (ASAH1, CATHL1, MYD88, and SEC61G) were higher in both PTB1 and PTB2 clusters and in all PTB samples analyzed together. Among these, MYD88 and SEC61G were similar in the two PTB clusters, while CATHL1 and ASAH1 were more abundant in PTB2.

**Figure 3:**
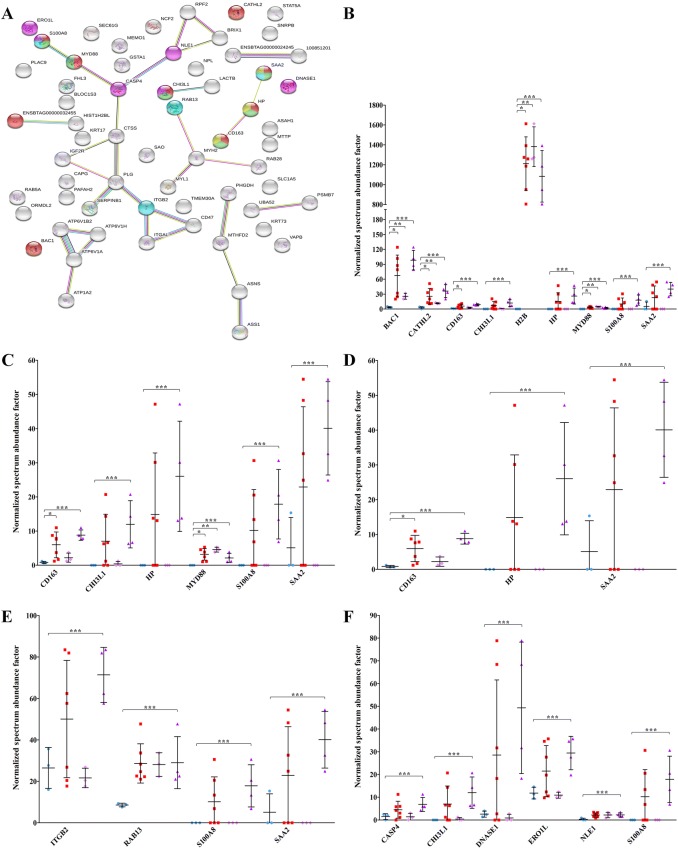
**GO analysis (biological process) of proteins significantly increased in PTB vs K.** The figure reports the protein network and the proteins included in each category with the respective abundance increases in all PTB samples, in PTB1, and in PTB2, respectively. **A** Protein network according to STRING. Proteins associated with defense response, inflammatory response, acute phase response, cell chemotaxis, and apoptotic process, are reported in red, green, yellow, sky blue, and fuchsia, respectively. Seven different colored lines link nodes and represent seven types of evidence used in predicting associations. Green lines: neighborhood evidence; red lines: presence of fusion evidence; blue lines: co-occurrence evidence; black lines: co-expression evidence; purple lines: experimental evidence; light blue lines: database evidence; yellow lines: text-mining evidence. Plots show the relative abundances of proteins in defense response (**B**), in inflammatory response (**C**), acute phase response (**D**), cell chemotaxis (**E**), and apoptotic process (**F**), indicating values obtained in K (sky blue), PTB (red), PTB1 (pink), and PTB2 (purple) samples. Asterisks indicate statistically significant differences between PTB vs K (*), PTB1 vs K (**), and PTB2 vs K (***) according to the t-test (*p* value ≤ 0.05). For protein identities corresponding to gene names, please see Table 1.

GO analysis (Figure 3A) highlighted 5 main networks. The first network was defense response (*P* = 8.65E−05) with CATHL1A, cathelicidin 2 (CATHL2), scavenger receptor cysteine-rich type 1 protein M130 (CD163), chitinase-3-like protein 1 (CHI3L1), histone H2B type 1 (H2B), haptoglobin (HP), MYD88, protein S100-A8 (S100A8), and serum amyloid A protein (SAA2) (Figure 3B). The second network was inflammatory response (*P* = 5.61E−04) with CD163, CHI3L1, HP, MYD88, S100A8, and SAA2 (Figure 3C). The third network was acute phase response (*P* = 3.47E−03) with CD163, HP, MYD88, and SAA2 (Figure 3D). The fourth network was cell chemotaxis (*P* = 2.25E−03) with ITGB2, ras-related protein Rab-13 (RAB13), S100A8, and SAA2 (Figure 3E). Finally, the fifth network was apoptotic process (*P* = 2.12E−02) with caspase-13 (CASP4 or CASP13), CHI3L1, deoxyribonuclease-1 (DNASE1), ERO1-like protein alpha (ERO1L or ERO1A), notchless protein homolog 1 (NLE1), and S100A8 (Figure 3F). These are described by a total of 15 proteins, 11 of which are significant only in the PTB2 cluster. As evidenced by KEGG enrichment analysis and GO analysis, proteins higher in PTB2 were likely associated with a more marked tissue alteration when compared to PTB1 and were characterized by numerous processes associated with the presence of MAP.

**Figure 4:**
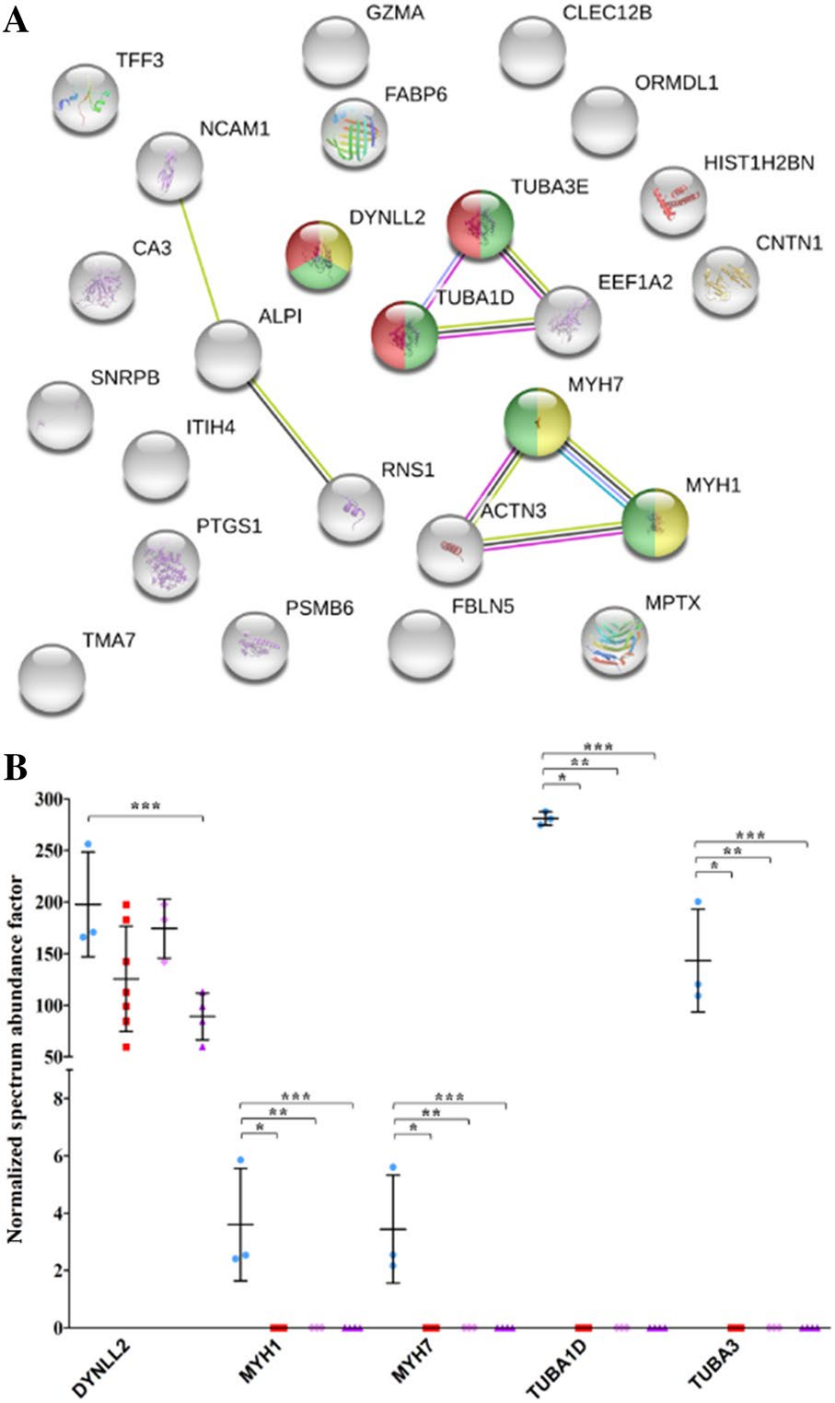
**GO analysis (cellular component) of proteins significantly decreased in PTB vs K.** The figure reports the protein network and proteins included in the category with the respective abundance decreases in PTB samples, in PTB1, and in PTB2, respectively. **A** Network obtained by STRING. Proteins associated with cytoskeleton, myosin complex, and microtubule are reported in green, yellow, and red, respectively. Seven different colored lines link nodes and represent seven types of evidence used in predicting associations. Green lines: neighborhood evidence; red lines: presence of fusion evidence; blue lines: co-occurrence evidence; black lines: co-expression evidence; purple lines: experimental evidence; light blue lines: database evidence; yellow lines: text-mining evidence. **B** Plot illustrating the abundance of proteins in the cytoskeleton network obtained in K (sky blue), PTB (red), PTB1 (pink), and PTB2 (purple) samples. Asterisks indicate statistically significant differences between PTB vs K (*), PTB1 vs K (**), and PTB2 vs K (***) according to the t-test (*p* value ≤ 0.05). For protein identities corresponding to gene names, please see Table 1.

The 27 proteins decreased in PTB (Figure 4A) were mainly involved in the networks biological process (*P* = 3.48E−02) and metabolic process (*P* = 3.48E−02) (Additional file 4). STRING revealed changes associated to cytoskeleton network (*P* = 1.17E−02), including dynein light chain 2 (DYNLL2), myosin-1 (MYH1), myosin-7 (MYH7), tubulin alpha-1D chain (TUBA1D), and tubulin alpha-3 chain (TUBA3E) (Figure 4B). These proteins are also found in the networks: myosin complex (*P* = 6.93E−04) including DYNLL2, MYH1 and MYH7, and microtubule (*P* = 3.05E−02) with DYNLL2, TUBA1, and TUBA3E.

### IHC evaluation of selected differential proteins

**Figure 5:**
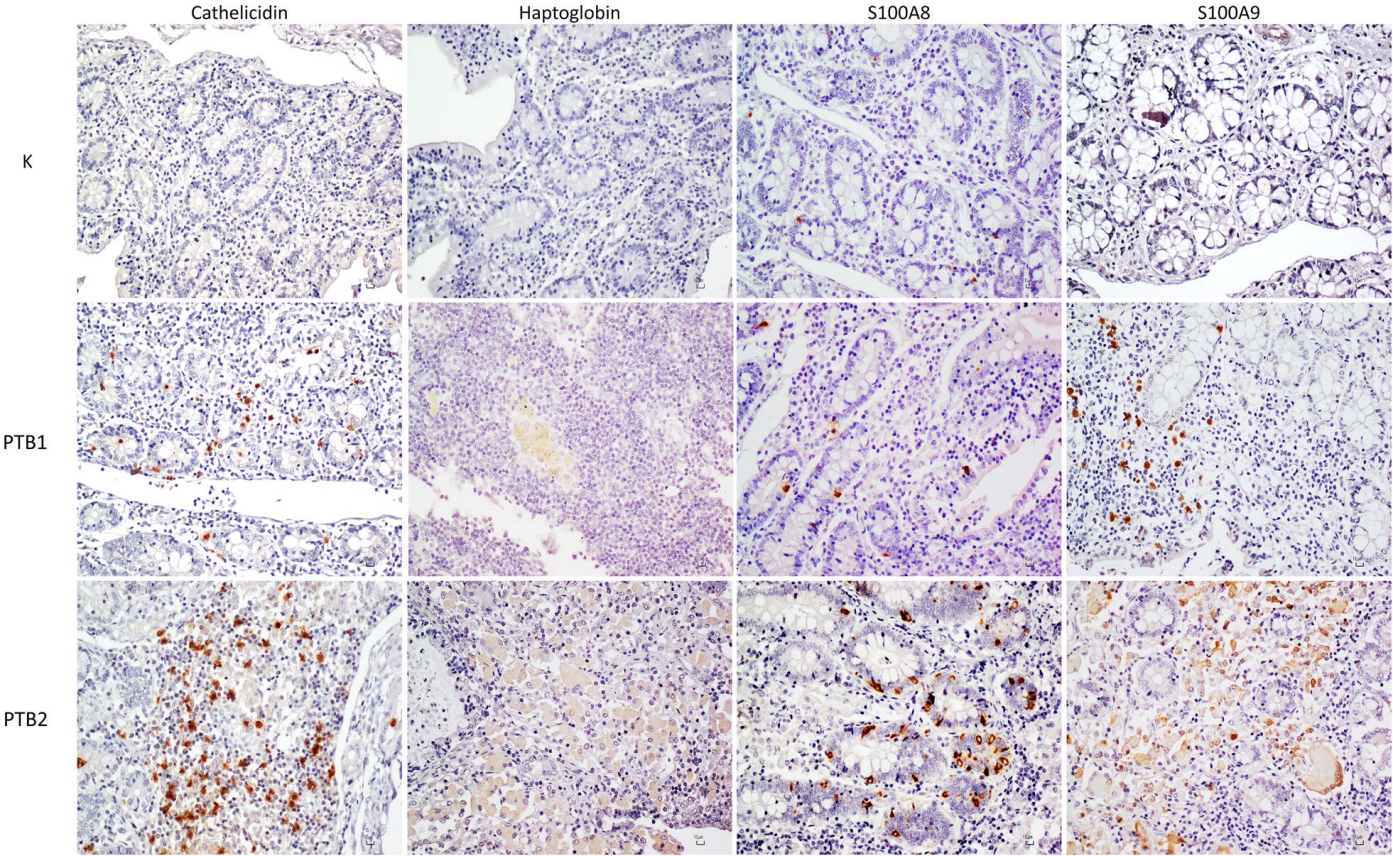
**Immunohistochemistry results for selected differential proteins.** Representative immunohistochemistry (IHC) images. First row. IHC results for negative control samples (K−). Second row. IHC results for PTB1 samples. Third row. IHC results for PTB2 samples.

Cathelicidin, haptoglobin, S100A8 and S100A9 were further investigated by IHC to validate proteomic results and to explore their value as potential indicators of disease severity or progression (Figure 5), in view of the observed differences in protein levels among samples and of their role in MAP infection and host immunity. Specifically, cathelicidin was selected because two different proteoforms (cathelicidin-1 and cathelicidin-2) were among the top 15 significantly higher proteins in PTB samples (Table 1), and also in consideration of its well-known role in mycobacterial killing [39] and innate immunity [40, 41]. Haptoglobin and S100A8 were selected for being the first and the second protein significantly increased only in PTB2 (Table 1). S100A9 was also assessed for being a subunit of the intestinal inflammation marker calprotectin together with S100A8 [42]. Mean, standard deviation, median, interquartile ranges, and minimum–maximum values of the number of positive cells observed in a 200× magnification field are reported in Additional file 5 for each marker. As a result, K samples (Figure 5, first row) were always negative to cathelicidins and haptoglobin while S100A8 and S100A9 showed few scattered signals. Cells positive to all four proteins were readily detectable in PTB samples, with significantly lower numbers in PTB1 (Figure 5, second row) than PTB2 (Figure 5, third row). Cathelicidin-positive cells (Figure 5, first column) were about 30-fold higher in PTB2 (median value 1.00 in PTB1 vs 30.50 in PTB2, *P* < 0.0001) and were morphologically compatible with dendritic cells (DCs, stronger signals) or epithelial cells (weaker signals). Haptoglobin-positive cells (Figure 5, second column) were about 40-fold higher in PTB2 than PTB1 (median value 0.00 in PTB1 vs 41.50 in PTB2, *P* < 0.0001). Haptoglobin was localized exclusively in correspondence of macrophages. S100A8-positive cells (Figure 5, third column) were about threefold higher in PTB2 (median value 6.00 in PTB1 vs 17.00 in PTB2, *P* < 0.0001). The cell type positive to S100A8 was compatible with Paneth cells for localization and morphology. S100A9-positive cells (Figure 5, fourth column) were about sixfold higher in PTB2 (median value 6.00 in PTB1 vs 36.00 in PTB2, *P* < 0.001). In the case of S100A9, the positive cell type was not clearly recognizable in PTB1, while it was almost exclusively macrophages in PTB2.

## Discussion

Differential shotgun proteomics enables to investigate the changes occurring in the protein makeup of a tissue in a condition of interest, such as in a pathological state against the physiological state. The proteomic approach presents advantages and disadvantages in comparison to gene expression strategies. One advantage is the ability to truly evaluate the extent of protein abundance, going beyond the estimates based on gene expression. In fact, several proteins do not follow a strict relationship with gene transcription but are regulated at translational or post-translational level, as in the case of cathelicidins [38, 43]. Moreover, adding to changes in protein abundance, changes in protein localization can often be revealed including protein capture or release phenomena, provided that histological evaluations are carried out for further investigation or validation. On the other hand, the number of samples in a high-performance shotgun proteomics study is typically limited by analytical throughput and cost issues. In addition, the sensitivity of proteomics is not as high as transcriptomics, and some relevant low-abundance, transient mediators may not be detected, including cytokines.

In this study, the application of shotgun proteomics to paucibacillary MAP-infected sheep ileal tissues (PTB) and matched MAP-free controls (K) highlighted several host proteins and pathways that are altered by MAP upon infection. In addition, proteomic results and histopathological grading revealed the presence of two PTB sample clusters, one with a higher (PTB2) and one with a lower proteomic divergence (PTB1) from K. That is, several proteins associated with MAP infection and with its pathological process changed more intensely in the PTB2 than in the PTB1 sample cluster. This was also reflected by the different abundance of the MAP chaperone protein HtpG in the two clusters, with FC values of 5.32 in PTB1 and 11.19 in PTB2, respectively. Based on histological observations, the PTB1 and PTB2 proteomic profiles might relate to paucibacillary type 2 and type 3a lesions [22], indicating that the different levels of tissue alteration and lesion severity found in sheep with subclinical, active, paucibacillary MAP infection might be associated to specific changes in their proteomic profiles.

According to KEGG enrichment analysis, the differential proteins seen in intestinal tissues of PTB sheep were mainly involved in phagosome formation, lysosome function and tuberculosis. This is due to the MAP strategy of survival within macrophages: impairment of phagosome and lysosome fusion. The mechanism is shared with other mycobacteria including the more intensively investigated *M. tuberculosis* [10]. Interestingly, most of these proteins changed significantly only in PTB2. Three out of eight proteins of the phagosome pathway were subunits of the V-type proton ATPase complex (V-ATPase). V-ATPase is responsible for phagolysosome acidification. By specifically excluding V-ATPase from this compartment, mycobacteria can survive and multiply within macrophages [10, 44]. Different authors have reported a higher expression of V-ATPase in MAP-infected macrophages as compared to macrophages infected with non-pathogenic mycobacteria [45–47]. This finding is in line with our previous work on multibacillary PTB, where we observed an increase in most V-ATPase subunits. In that work, consistently with a higher MAP load and lesion severity, fold changes were very high for all subunits and peaked up to a FC of 18.09 for subunit H, the specific target for mycobacterium-mediated exclusion [25, 44].

Changes in Rab GTPases were also observed. It is known that Rab5 stimulates fusion of early endosomes while Rab7 promotes fusion of mature phagosomes with endosomes and lysosomes [48]. By retaining Rab5, MAP impairs maturation of endosomes into functional mycobactericidal compartments [49]. In line with this, we observed that Rab5 was significantly increased in the PTB2 sample cluster (but not in PTB1). On the other hand, Rab7 was unchanged in both clusters, highlighting the role of Rab5 in the mycobacterial pathogenicity mechanism. The increase seen in Rab13 does also relate to the relevance of the phagocytotic pathway in the host defense from MAP. Tubulins are involved in phagosome maturation, and their massive decrease seen in infected tissues might be related to the impairment of this process in MAP-infected macrophages.

According to GO analysis, most proteins undergoing significant changes in MAP-infected tissues were involved in defense response, inflammatory response and acute phase response. Two isoforms of cathelicidin were increased in all PTB samples, with higher FC values in PTB2 vs PTB1. This result was also in line with our previous study on multibacillary PTB, (FCs of 24.53 for cathelicidin 1 and of 15.79 for cathelicidin 2) [25]. Cathelicidins are well-known antimicrobial peptides with a crucial role in the intracellular killing of mycobacteria in macrophages [39, 50]. Besides their direct antimicrobial functions, cathelicidins play multiple roles as inflammation mediators [51], and their importance in the innate immune defense of ruminant is highlighted by the unusually large number of genes compared to a single copy in most other mammalians, such as humans and mice [52]. Consistently with the proteomic data, IHC confirmed the absence of cathelicidin in K tissues and its presence in PTB tissues, with a significantly higher abundance in PTB2 vs PTB1. However, it was not possible to specifically identify the cathelicidin isoform(s) by IHC due to the polyclonal anti-cathelicidin antibodies used. Apparently, DCs were responsible for most of the cathelicidin signal; however, such DC localization might be due to protein capture rather than to protein expression by DCs themselves. Based on studies in humans, cathelicidin (LL-37) is internalized by DCs with subsequent localization primarily in the cytoplasmic compartment and then in the nucleus. This eventually leads to a suppression of their response to toll-like receptor ligands and renders DCs less capable to activate T lymphocytes. In other words, cathelicidins inhibit DC function [51, 53, 54]. Nevertheless, in human tuberculosis this is inverted by presence of vitamin D, in a finely regulated equilibrium between inhibition and stimulation of cathelicidin production, DC regulation and Th1 differentiation [55]. This is only part of the puzzle governing the combined action of genetic background, farming variables and environmental conditions (such as nutritional balance and exposure to light) in resistance of ruminants to mycobacterial diseases, in which the role of cathelicidin regulation of DCs might deserve further investigation.

Several proteins involved in the acute phase response were significantly changed in PTB vs K. Interestingly, haptoglobin, S100A8 and serum amyloid protein A were seen only in PTB2 samples and were not detected in PTB1 or K. This might underscore a potential role for these proteins as markers of disease progression, possibly indicating the activation of an acute inflammatory process when MAP infection is less controlled by the host. Of note, haptoglobin showed the highest increase among all the proteins significant only in PTB2 vs K, with a FC of 13.84. In our previous study on multibacillary MAP infection, haptoglobin showed a FC of 22.93 [25]. Here, upon IHC validation, haptoglobin was abundantly present in tissues of the PTB2 cluster while it was not detected in tissues of the PTB1 cluster. Notably, haptoglobin-positive cells appeared to be almost exclusively macrophages. This might be related to its scavenging functions; in fact, haptoglobin captures hemoglobin by forming a complex that is promptly internalized by macrophages. Its endocytosis and targeting to the lysosome are mediated by the macrophage receptor CD163 (scavenger receptor cysteine-rich type 1 protein M130) [56]. Consistently with this observation, CD163 was significantly increased only in the PTB2 Cluster. Once again, the observed increase in protein abundance within the affected tissue is probably due to selective capture, rather than increased expression, by the cell. An increase in the CD163 receptor has been observed also in ileal tissues of cattle infected by MAP [57]. It would be interesting to investigate how an increased availability of iron within the macrophage phagosomes is reflected on MAP pathogenicity.

S100A8 plays a prominent role in the regulation of inflammation and immune response. It can induce neutrophil chemotaxis and adhesion, and it is predominantly found as the calprotectin heterodimer in association with S100A9, with a wide plethora of intra- and extracellular functions [58]. Fecal calprotectin is currently used in human medicine to differentiate organic from functional bowel disorders [42]. Upon IHC validation, S100A8 was detected in all PTB samples, but few, scattered positive cells were also present in K tissues. The number of S100A8-positive cells was about three times higher in PTB2 than PTB1. In consideration of its known association with S100A9, it was surprising that the latter protein was not detected by proteomics in any of the experimental samples. However, S100A9 was readily detected by IHC in all PTB samples. The number of S100A9-positive cells was higher than that of S100A8-positive cells, and the cell type was also different. A possible explanation is that S100 proteins can also exist as homodimers [58], and a differential expression or a selective capture of S100A9 by a specific cell type might be occurring.

Another protein significantly increased in PTB was protein transport protein Sec61 (SEC61). As recently discovered, SEC61 is present in antigen-containing endosomes of DCs after stimulation with microbial substances and is essential for endosome-to-cytosol translocation and for antigen cross-presentation to T cells [59]. This pathway enables DCs to present extracellular antigens onto MHC I molecules, thereby stimulating naive cytotoxic CD8+ T cells into activated cytotoxic CD8+ T cells. This is only part of the complex mechanisms responsible for the predominance of the Th1 or Th2 response, a crucial step in the control of mycobacterial infections.

H2B was the most increased protein in all PTB samples. This might relate to the well-known antimicrobial role of histones. On the other hand, ubiquitin was the second highest protein, and this might indicate the occurrence of an extensive histone ubiquitination [60]. Nevertheless, it should be reminded that shotgun proteomics results as analyzed and presented here can only provide information on relative protein abundance. These data can give useful hints on protein networks and pathways activated upon infection, but indications on protein structure, protein interactions or post-translational modifications need dedicated experimental investigations or data analysis algorithms.

Adding to proteins increased in PTB2, proteins significantly increased only in samples of the PTB1 cluster (such as fatty acid-binding protein, glutathione S-transferase, Na/K-transporting ATPase, argininosuccinate synthase) or detected only in the PTB1 cluster (such as Rab-28), could also be worth investigating further; these proteins might represent potential indicators of a better ability to contrast disease and, should this be demonstrated, have potential as targets in genetic selection for resistance to mycobacterial diseases.

Finally, as anticipated, it should be reminded that this study was carried out on a limited number of animals due to methodological constraints. Stringent statistic procedures were applied in differential proteomic analysis and IHC validation confirmed the main study findings, but observations such as the relationship of PTB1 and PTB2 clusters with type 2 and type 3a lesions or any hypothesis on their connection with disease progression or increased pathogenicity and associated pathways will need to be investigated further with studies on a larger, well-characterized sample cohort by means of higher throughput techniques, such as IHC or other molecular tests, for a thorough validation on the entire range of lesions including also multibacillary cases. It should also be highlighted that low-abundance mediators such as cytokines were not identified in this study, as in most shotgun proteomics studies. However, these have been investigated in detail by other authors in paucibacillary PTB [17, 20, 61, 62].

In conclusion, this work provided the first detailed proteomic characterization of paucibacillary MAP-infected sheep ileal tissues in comparison to MAP-free tissues. The differential proteomic analysis combined with immunohistochemical validation highlighted several changes occurring in PTB tissues and provided novel molecular information on the distinct levels of tissue involvement that can be found within the asymptomatic, paucibacillary condition. Adding to useful insights into disease processes and pathways, we identified several proteins that are absent in healthy tissues and are present in MAP-infected tissues, with differences in abundance levels or localization possibly reflecting different degrees of lesion severity. These host protein markers can assist future investigations aimed at understanding PTB evolution and progression, either with classical post-mortem analysis of tissues or, hopefully, by enabling the development of immunoassay tools for their in vivo detection in feces.

## Additional files

**Additional file 1.** **Histopathological grading scores and histopathological features of the PTB1 and PTB2 cluster samples.** The file describes in detail: i) the grading scores assigned to all sheep tissue samples considered in this study; ii) Hematoxylin–Eosin and Ziehl–Neelsen stained panoramic and detailed views of PTB1 and PTB2 tissues illustrating their main features.

**Additional file 2.** **Number of identified proteins, peptides, PSMs and Search Inputs in K and P samples.** The file contains a table with the detailed report of all the results obtained for each tissue sample, and the average values measured in K and P samples.

**Additional file 3.** **Protein identification details and differential proteomic results based on label-free quantitation.** Excel file reporting: the list of all proteins identified in each sample with the detailed data for coverage, proteins, unique peptides, and PSMs; the average NSAF value of each sheep protein in all experimental samples; the average NSAF value of each MAP protein in all experimental samples; and the complete list of all differential proteins detected in PTB vs K, PTB1 vs K, and PTB2 vs K, with the respective R_SC_, FC, beta-binomial p value, and beta-binomial p value after Benjamini–Hochberg correction.

**Additional file 4.** **Results of KEGG enrichment analysis and GO analysis.** Excel file reporting the detailed results obtained with STRING for KEGG enrichment analysis, Biological process, Molecular function and Cellular component.

**Additional file 5.** **Column statistics of IHC results.** Table reporting the mean, standard deviation (SD), median, interquartile range (IQR), minimum and maximum (Min–Max) value of the number of cells measured in a 200× magnification field of the distal ileum in PTB1 and PTB2 sheep.
