# Supplementary material for: Characterization of paucibacillary ileal lesions in sheep with subclinical active infection by Mycobacterium avium subsp. paratuberculosis
Source: Vet Res. 2018 Dec 4;49:117. doi: 10.1186/s13567-018-0612-0 (PMC6278003; doi:10.1186/s13567-018-0612-0)

**Additional file 1 Histopathological grading scores.** A score from 0 to 3 was assigned based on feature intensity. K: controls; P: paucibacillary PTB samples from the 7 sheep.

| **Parameter** | **K1** | **K2** | **K3** | **P1** | **P2** | **P3** | **P4** | **P5** | **P6** | **P7** |
| --- | --- | --- | --- | --- | --- | --- | --- | --- | --- | --- |
| **Ziehl-Neelsen** | 0 | 0 | 0 | 1 | 2 | 1 | 0 | 1 | 0 | 0 |
| **PCR** | 0 | 0 | 0 | 2 | 2 | 2 | 1 | 2 | 1 | 1 |
| **Mucosa lesions** | 0 | 0 | 0 | 1 | 2 | 1 | 1 | 2 | 1 | 1 |
| **Submucosa lesions** | 0 | 0 | 0 | 1 | 2 | 1 | 0 | 1 | 0 | 0 |
| **Oedema of submucosa** | 0 | 0 | 0 | 1 | 1 | 1 | 0 | 0 | 0 | 0 |
| **Erosion-fusion of villi** | 0 | 0 | 0 | 2 | 2 | 2 | 0 | 1 | 0 | 0 |
| **Granulomas** | 0 | 0 | 0 | 0 | 2 | 1 | 0 | 2 | 1 | 1 |
| **Macrophages** | 0 | 0 | 0 | 2 | 3 | 1 | 1 | 2 | 1 | 1 |
| **Epithelioid macrophages** | 0 | 0 | 0 | 3 | 3 | 2 | 1 | 2 | 1 | 1 |
| **Giant cells** | 0 | 0 | 0 | 0 | 2 | 0 | 0 | 1 | 0 | 0 |
| **Lymphocytes** | 0 | 0 | 0 | 2 | 1 | 2 | 2 | 2 | 2 | 1 |
| **Eosinophils** | 0 | 0 | 0 | 1 | 0 | 0 | 1 | 1 | 2 | 2 |
| **Plasmacells** | 0 | 0 | 0 | 1 | 0 | 0 | 0 | 1 | 1 | 1 |
| **Oedema of villi** | 0 | 0 | 0 | 1 | 2 | 1 | 1 | 2 | 1 | 1 |
| **Necrosis** | 0 | 0 | 0 | 0 | 0 | 0 | 0 | 0 | 1 | 1 |
| **Pyknosis of nuclei** | 0 | 0 | 0 | 0 | 0 | 0 | 0 | 1 | 0 | 0 |

Table legend: 0, negative; 1, rare; 2, moderate; 3, high.

**Histopathological features of the PTB1 and PTB2 cluster samples.**

Top rows: PTB1. A: panoramic view of terminal ileum. The image highlights a slightly erosion of villi, active Peyer’s patches protruding up to the middle of the mucosa, and several eosinophilic regions. B, C, D; representative features in Hematoxylin-Eosin stained tissues; a granuloma composed of epithelioid macrophages (black arrows) in Peyer’s patch (B); lymphocytes (red stars) and eosinophilic cells (black stars) widespread in mucosa (C); epithelioid macrophages (black arrow), rare lymphocytes and eosinophilic cells in the apex of villi (D). E, F, G: representative images of Ziehl-Neelsen stained tissue; no AFB are visible in the macrophages of Peyer’s patch (E) and in the apex of villi (F, G).

Bottom rows: PTB2. A, B: panoramic views of terminal ileum. The images highlight erosion of villi and submucosal edema. C, D, E: representative features in Hematoxylin-Eosin stained tissues; giant cells (black arrow) (C); lymphocytes (red stars), and plasma cells (black stars) in the middle of mucosa (D); epithelioid macrophages (red stars), and eosinophilic cells (black stars) in apex of the villi (E); F, G, H; representative fields of Ziehl-Neelsen stained tissue; few AFB-positive areas can be seen in epithelioid macrophages in Peyer’s patches and in the apex of villi.


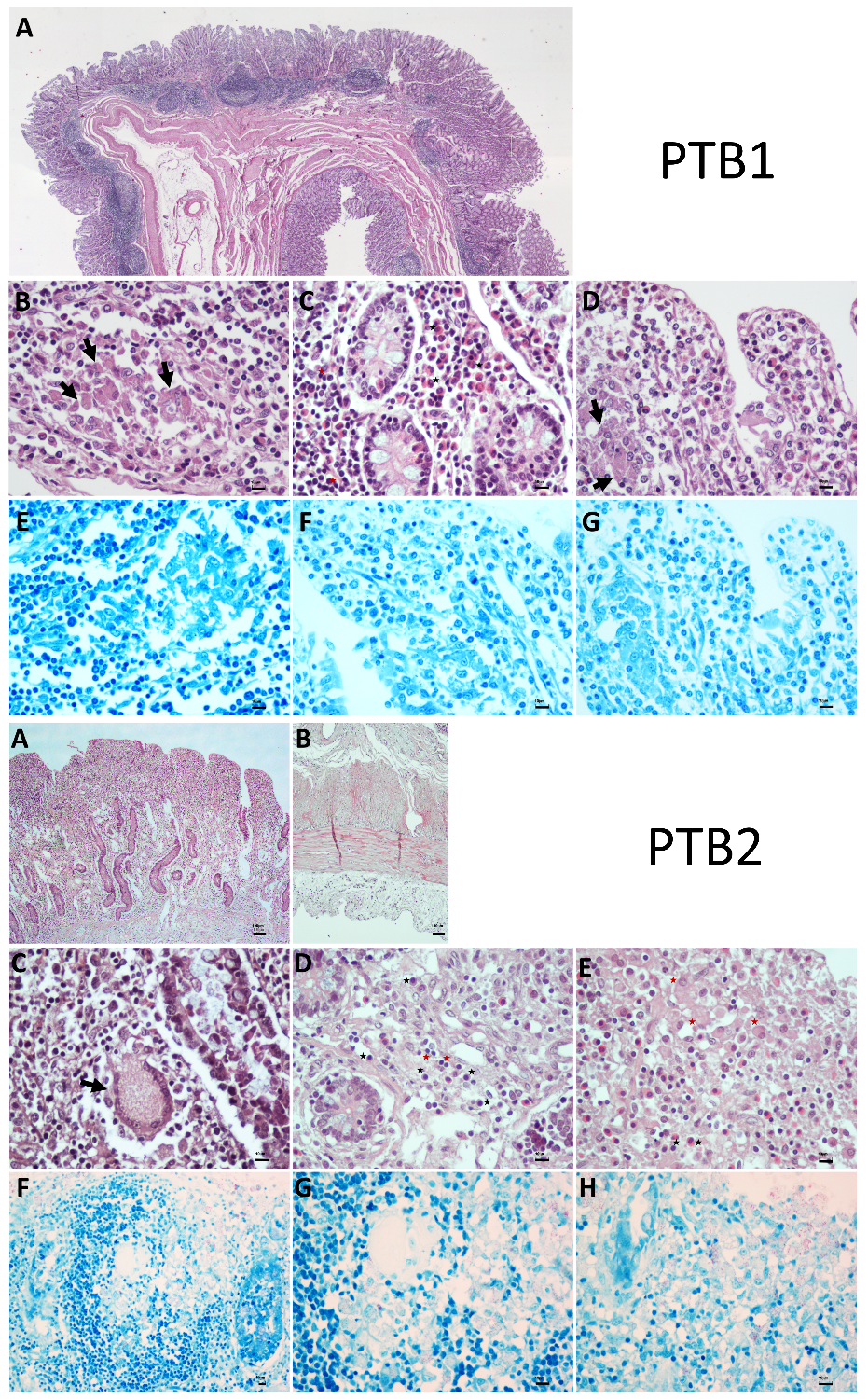

Supplement: Supplementary file 1 — Additional file 1. Histopathological grading scores and histopathological features of the PTB1 and PTB2 cluster samples. The file describes in detail: i) the grading scores assigned to all sheep tissue samples considered in this study; ii) Hematoxylin–Eosin and Ziehl–Neelsen stained panoramic and detailed views of PTB1 and PTB2 tissues illustrating their main features. [file 13567_2018_612_MOESM1_ESM.docx]
