# Supplementary material for: Characterization of paucibacillary ileal lesions in sheep with subclinical active infection by Mycobacterium avium subsp. paratuberculosis
Source: Vet Res. 2018 Dec 4;49:117. doi: 10.1186/s13567-018-0612-0 (PMC6278003; doi:10.1186/s13567-018-0612-0)
Supplement: Supplementary file 2 — Additional file 2. Number of identified proteins, peptides, PSMs and Search Inputs in K and P samples. The file contains a table with the detailed report of all the results obtained for each tissue sample, and the average values measured in K and P samples. [file 13567_2018_612_MOESM2_ESM.docx]

**Additional file 2 Number of identified proteins, peptides, PSMs and Search Inputs in K and P samples.**

| **Samples** | **Proteins** | **Peptides** | **PSMs** | **Search Inputs** |
| --- | --- | --- | --- | --- |
| K1 | 2430 ± 41 | 14 163 ± 118 | 34 765 ± 972 | 223 331 ± 2250 |
| K2 | 2256 ± 77 | 12 924 ± 279 | 31 483 ± 796 | 216 991 ± 5125 |
| K3 | 2190 ± 29 | 12 156 ± 116 | 30 379 ± 401 | 205 300 ± 383 |
| **Average K** | **2292 ± 124** | **13 081 ± 1012** | **32 209 ± 2282** | **215207 ± 9147** |
| P1 | 2189 ± 6 | 12 879 ± 105 | 26 243 ± 13 | 203 937 ± 155 |
| P2 | 2540 ± 6 | 16 377 ± 378 | 35 542 ± 236 | 211 510 ± 2784 |
| P3 | 2092 ± 7 | 12 216 ± 93 | 26 565 ± 256 | 204 311 ± 100 |
| P4 | 2481 ± 6 | 15 254 ± 143 | 37 378 ± 126 | 221 176 ± 188 |
| P5 | 2519 ± 25 | 16 434 ± 245 | 39 010 ± 278 | 211 691 ± 619 |
| P6 | 2312 ± 33 | 14 615 ± 355 | 27 937 ± 526 | 213 125 ± 334 |
| P7 | 2317 ± 18 | 14 209 ± 52 | 32 384 ± 129 | 204 948 ± 165 |
| **Average P** | **2350 ± 172** | **14 569 ± 1620** | **32 151 ± 5320** | **210 099 ± 6250** |

Numbers represent means ± SD for all samples.
