# Supplementary material for: Characterization of paucibacillary ileal lesions in sheep with subclinical active infection by Mycobacterium avium subsp. paratuberculosis
Source: Vet Res. 2018 Dec 4;49:117. doi: 10.1186/s13567-018-0612-0 (PMC6278003; doi:10.1186/s13567-018-0612-0)
Supplement: Supplementary file 5 — Additional file 5. Column statistics of IHC results. Table reporting the mean, standard deviation (SD), median, interquartile range (IQR), minimum and maximum (Min–Max) value of the number of cells measured in a 200× magnification field of the distal ileum in PTB1 and PTB2 sheep. [file 13567_2018_612_MOESM5_ESM.docx]

**Additional file 5 Column statistics of IHC results.** The table reports the mean, standard deviation (SD), median, interquartile range (IQR), minimum and maximum (Min-Max) value of the number of cells measured in a 200X magnification field of the distal ileum in PTB1 and PTB2 sheep.

| **Marker** | **Mean** | **SD** | **Median** | **IQR** | **Min-Max** |
| --- | --- | --- | --- | --- | --- |
| Cathelicidin PTB1 | 2.433 | 3.036 | 1.000 | 0.00-3.250 | 0.00-12.00 |
| Cathelicidin PTB2 | 33.80 | 24.55 | 30.50 | 11.00-52.50 | 1.00-87.00 |
| Haptoglobin PTB1 | 1.367 | 4.351 | 0.0 | 0.00-0.00 | 0.00-19.00 |
| Haptoglobin PTB2 | 47.28 | 45.57 | 41.50 | 3.25-85.50 | 0.00-156.00 |
| S100A8 PTB1 | 7.533 | 5.758 | 6.000 | 3.750-10.25 | 0.00-29.00 |
| S100A8 PTB2 | 18.90 | 9.755 | 17.00 | 12.00-25.00 | 0.00-45.00 |
| S100A9 PTB1 | 21.47 | 28.55 | 6.00 | 2.00-33.50 | 0.00-94.00 |
| S100A9 PTB2 | 49.28 | 38.24 | 36.00 | 17.25-72.50 | 5.00-148.00 |
